# Nocturia as a clinical indicator of severe obstructive sleep apnea syndrome and its response to CPAP or surgical treatment

**DOI:** 10.5935/1984-0063.20220067

**Published:** 2022

**Authors:** Alberto Labra, Montserrat Roldan-Navarro, Reyes Haro-Valencia, Francisco Sánchez-Narvaez, Mauricio Ruiz-Morales

**Affiliations:** 1 Mexican Institute of Integral Sleep Medicine, Otolaryngology - Mexico City - Mexico; 2 Mexican Institute of Integral Sleep Medicine, CPAP Clinic - Mexico City - Mexico; 3 Mexican Institute of Integral Sleep Medicine, Director - Mexico City - Mexico; 4 Mexican Institute of Integral Sleep Medicine, Clinical Research - Mexico City - Mexico

**Keywords:** Obstructive Sleep Apnea, Nocturia, Surgical Procedures

## Abstract

**Introduction:**

Obstructive sleep apnea syndrome (OSAS) is a common condition that has been associated to a number of metabolic, cardiovascular and cognitive consequences. Its diagnosis relies on a polysomnographic or polygraphic study, but clinical findings remain as an important part of the diagnostic process. Nocturia is a common symptom that may indicate severe OSAS, but it is often forgotten in the initial evaluation of these kind of patients. Positive airway pressure (CPAP) is known to reduce nocturia, but the roll of surgery is not clear about it.

**Material and Methods:**

A case series is presented. We compare 2 groups of male adults with severe OSAS, the first group treated with CPAP for 3 months, while group 2 underwent a multilevel surgical management. Apnea-hypopnea index (AHI) and the nocturia events number (NEN) were assessed before and after the treatment. Frequencies, descriptive statistics and a related sample Student’s *t*-test were performed for statistical analysis.

**Results:**

97 male patients were included, age ranged from 29 to 71 years old. In group 1, treated with CPAP, AHI mean was 54.59 and nocturia mean 4.53 before treatment. With CPAP, the AHI mean was 6.63 and NEN mean 0.51. In group 2, AHI mean before surgery was 40.02, NEN mean 3.78, and after the surgical management AHI mean was 7.74 and NEN mean 0.7. Student’s *t*-test in groups 1 and 2 showed a *p*=0.000.

**Conclusions:**

AHI and NEN were clearly related in both groups, and the improvement of NEN and AHI were consistent in both groups, the CPAP and the surgical treatment. The presence of a NEN value of 4-5 may be an indicator of severe OSAS and should always be clinically evaluated.

## INTRODUCTION

Obstructive sleep apnea syndrome (OSAS) diagnosis is a complex issue in sleep medicine, and it should always include its common symptoms: snoring, excessive daytime somnolence, asthenia, poor work performance, the history of stroke or arterial hypertension and a polysomnography. Its treatment is complex as well; it may include surgery or medical approaches, being the use of continuous positive airway pressure (CPAP) the gold standard^1^.

Once the diagnosis is set, it is mandatory to identify the anatomical areas involved within the upper airway, from the nose to the larynx. The physical examination should be able to rule out if the narrowing of the upper airway is related to obstructive structures or to collapse of the pharyngeal walls, in order to determine the best treatment option for each patient^2^.

Nevertheless, there are some other symptoms associated to OSAS, but in the clinical setting, we may not always be aware of them in order to make a deeper research. One of those symptoms is nocturia. It is defined as “the need to get up once or several times per night for nocturnal urination” and it is present in nearly half of the OSAS patients, but its prevalence is higher in snoring men. There a number of associated conditions linked to the development of nocturia, such as cardiovascular disease, hypertension, diabetes mellitus, prostatic conditions, lower urinary tract obstruction and infection, and some environmental factors^3^.

Nocturia may be present in non-OSAS subjects, but its prevalence is significantly higher in OSAS patients than in healthy individuals (70% vs. 25%). Some authors have reported that in these patients there is an elevated atrial natriuretic peptide excretion, so this might be the related pathophysiologic mechanism generating nocturnal voids^4^. Other pathophysiologic mechanisms proposed to explain the need to urinate are a decreased renin-angiotensin-aldosterone secretion and exaggerated intrathoracic pressure swings due to the sleep apnea.

OSAS clinical features tend to stimulate the production of ANP, thus having an increased amount of nocturia events. It has been reported that CPAP treatment of OSAS is related to a decrease and control of nocturia, but there are no reports of the effect of sleep apnea surgery on ANP levels and nocturia events. The main objective of this study is to determine whether severe OSAS patients suffer of nocturia consistently and our second objective is to determine if upper airway surgery on these patients is also useful to decrease the nocturia events number in patients with severe sleep apnea.

## MATERIAL AND METHODS

We present a case series of consecutive adult patients with severe obstructive sleep apnea syndrome. All of them were diagnosed and treated by the authors. The study was approved by the ethics and research committee, number IMMIS 2021/02, and the patients signed an informed consent.

Uncontrolled diabetes mellitus, prostatic hypertrophy, uncontrolled hypertension, lower urinary tract diseases, and other causes of nocturia were assessed and discarded during the initial clinical evaluation.

The patients were divided into two groups. Group 1 were treated with CPAP for 3 months, these patients were advised to use the positive pressure therapy 7 days per week, for at least 6 hours per night. Adherence to positive pressure treatment was assessed using the data recorded at the CPAP device. Group 2 were candidates for surgical treatment: body mass index <30, neck circumference <16”, with clearly obstructive structures at the nose and pharynx (nasal septum, turbinates, tonsils, adenoids, soft palate, uvula, tongue, and epiglottis). All of these patients underwent clinical physical assessment as well as supine endoscopy. Drug induced sleep endoscopy (DISE) was performed in those patients in whom the obstruction levels were not easily found.

The CPAP titration was performed as a part of a split night polysomnography. All of the surgically treated patients underwent a multilevel approach of the upper airway, including nasal (septoplasty and turbinate procedures) and pharyngeal procedures (base of tongue, palate, tonsils and epiglottis were addressed according to the obstructive anatomical level of each particular patient). It should be noted that surgery was individualized according to the anatomical characteristics of each patient. A control polysomnography was performed 3 months after the surgical or positive airway pressure use, along with an extensive clinical evaluation.

We assessed the number of nocturia events (NEN) and the apnea-hypopnea index (AHI) before and after the treatment. Average NEN was assessed during the initial clinical interview and after 3 months of treatment only on a clinical basis. Nocturia is one of the questions in our routine evaluation questionnaire.

As for the statistical analysis, frequencies and descriptive statistics were performed. A related sample Student’s *t*-test was performed to compare the media of nocturia events and AHI before and after surgery or CPAP treatment. The statistical software PSPP 1.4.1 (GNU Project, 2020) was used. A *p*-level<0.005 was considered statistically significant. For sensibility and specificity, Epidat 3.0 software was used.

## RESULTS

A total of 97 male patients with severe OSAS (AHI>30) were included in this study. All of them underwent a split-night polysomnography with CPAP titration. Their age ranged from 29 to 71, with a media of 50.86 (standard deviation = 9.7). Before CPAP or surgical management, AHI average was 47.68 (standard deviation = 16.47), nocturia ranged from 2 to 7 events per night, for an average of 4.18 and standard deviation = 1.01. After the treatment, AHI average was 7.15 (standard deviation = 3.33), and nocturia events range was from 0-2 events, mean = 0.6 and standard deviation = 0.64. Complete frequency results can be found in Table 1.

**Table 1:** Complete apnea-hypopnea index (AHI) and nocturia events number (NEN) frequency values. Total sample = 97 patients, CPAP patients = 51, surgery patients = 46.

	Age (years old)	AHI pre-CPAP	AHI post-CPAP	AHI pre-surgery	AHI post-surgery	NEN pre-CPAP	NEN post-CPAP	NEN pre-surgery	NEN post-surgery
Media	50.86	54.59	6.63	40.02	7.74	4.53	0.51	3.78	0.7
Min	29	30	1	31	2	3	0	2	0
Max	71	108	14	56	14	7	2	6	2
Standard deviation	9.7	19.45	3.49	6.68	3.09	1.03	0.61	0.84	0.66

In the group of nonsurgical patients, pre-CPAP results were: IAH mean = 54.59 (standard deviation = 19.45), nocturia events mean = 4.53 (standard deviation = 1.03). The post-CPAP result were as follows: IAH mean = 6.63 (standard deviation = 3.49), and nocturia events mean = 0.51 (standard deviation = 0.61).

On the other hand, in the group of patients who underwent a multilevel surgery, pre-surgery results showed: IAH mean = 40.02 (standard deviation = 6.68) and nocturia events mean=3.78 (standard deviation=0.84). Post-surgery results were: IAH mean=7.74 (standard deviation=3.09) and nocturia events mean=0.7 (standard deviation=0.66).

Student’s *t*-test for related samples in the group of CPAP patients showed, for the AHI a *t* value of 19.48 (95% confidence interval = 2.46-52.9) and *p*<0.000. For the nocturia events number, the *t* value was of 30.96 (95% confidence interval = 3.76-4.28), and *p*<0.000.

Finally, in the group of surgically treated patients, Student’s *t-*test showed as follows: AHI *t* value of 35.91 (95% confidence interval = 30.47-34.09) and *p*<0.000. And, for nocturia events, the *t* value was 22.3 (95% confidence interval = 2.81-3.37) and *p*<0.000 (Table 2).

**Table 2:** Student’s *t*-test for related samples in the group treated with CPAP and in the group treated with surgery.

	Nocturia pre/post-CPAP	AHI pre/post-CPAP	Nocturia pre/post- surgery	AHI pre/post- surgery
Media	4.02	47.96	3.09	32.28
Standard deviation	0.93	17.58	0.94	6.1
95% confidence interval	3.76-4.28	43.02-52.9	2.81-3.37	30.47-34.09
t	30.96	19.48	22.3	35.91
Degrees of freedom	50	50	45	45
2 tailed significance	0.000	0.000	0.000	0.000

**Figure 1:**
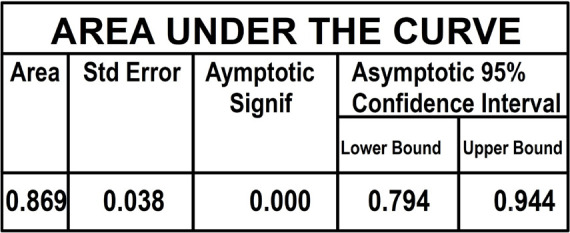
Once the area under the curve was calculated, a value of 0.869 was obtained, with a significant confidence interval range 0.794-0.944.

**Figure 2:**
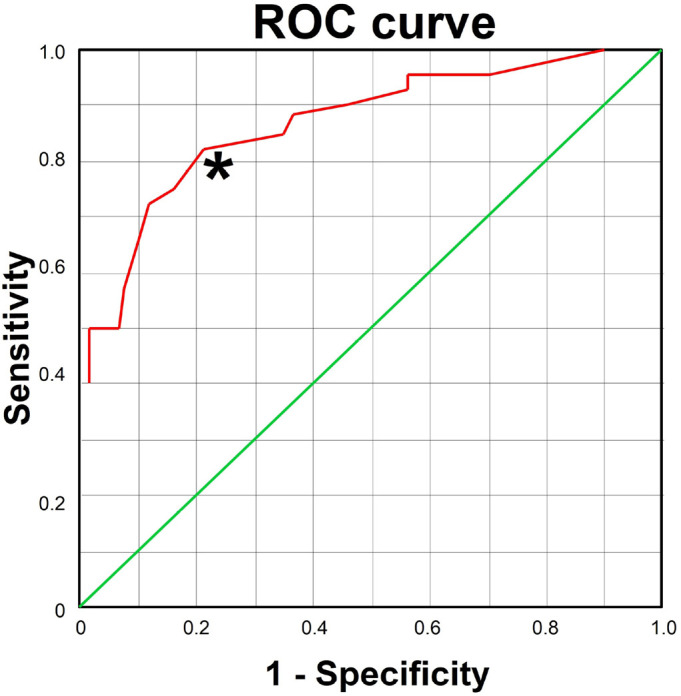
ROC curve representing sensitivity and specificity.

In order to get sensibility and specificity, patients were divided into quartiles, taking the 75 quartile, corresponding to a nocturia events number of 5 or more, and this value was contrasted with the AHI. Curve coordinates were obtained, considering the results of the ROC curve, we found a sensibility = 0.825 and specificity = 1-0.211 (0.789). Calculation of the area under the curve showed a value of 0.869 (0.794-0.944) (Figures 1 and 2).

## DISCUSSION

Despite nocturia is a well-known symptom that may help us to rule out the presence of sleep apnea, most of the sleep professionals often forget to deepen their assessment of this condition. A thorough clinical evaluation is mandatory to improve our diagnosis, it should always include as many symptoms as possible, and nocturia is one of them.

On the other hand, surgery remains as a very controversial kind of treatment for obstructive sleep apnea, especially in cases of severe OSAS. The indications for surgery are not always clear, and remain as one of the most controversial issues of sleep disordered breathing conditions.

Our study has some limitations that must be addressed. It is a retrospective study, where very specific data are compared, in order to keep internal validity. We only included severe OSAS because it is the diagnosis in most of the patients at our sleep disorders center.

Another weakness of this study is that nocturia was assessed only with one question, before and after the treatment. A sleep diary, where the patient can register nocturnal events may represent a good tool for future studies addressing nocturia.

The primary objective of our study was to verify whether patients with obstructive sleep apnea presented nocturia consistently. Given that all of our patients had severe OSAS, and that nocturia events number ranged from 2 to 7 with a mean of 4.8, we may consider that having 4-5 events of nocturia per night could be associated to severe OSAS, in patients in whom an exhaustive clinical diagnosis has been made, and of course, excluding any other possible cause of nocturia. However, this should be confirmed by comparing these results with mild and moderate OSAS patients. Due to the design of this study, we cannot claim there is a causal relationship.

Atrial natriuretic peptide (ANP), also called atrial natriuretic factor is a 28-aminoacid peptide, with a 17-aminoacid ring in the middle of the molecule. This peptide is synthesized and secreted by the cardiac muscle cells, especially in the walls of the cardiac atria. These myocytes contain volume receptors, which easily respond to the increased stretching of the cardiac walls associated to an increased atrial blood volume. It is also secreted in response to sympathetic stimulation of ß-receptors (which is common in the OSAS-related arousals), hypernatremia, hypertension, and hypoxemia^5,6^. The main effect of ANP is to keep the homeostasis in the volume regulation. In the kidneys, the ANP increases glomerular filtration rate, inhibits the effect of angiotensin II and the blood flow in renal circulation. ANP also decreases sodium reabsorption and inhibits renin secretion, thereby blocking the renin-angiotensin-aldosterone system. All these actions are aimed at the elimination of sodium through the urinary tract, thus increasing the production of urine. The ANP also inhibits cardiac hypertrophy, relaxes vascular smooth muscle in arterioles and reduces the aldosterone secretion by the adrenal cortex^7-10^.

On this series, all of the subjects were men, despite they were consecutive patients. This may seem biased, but it can be explained because one of our selection criteria was “severe OSAS” and the prevalence of severe apnea significantly lower in female patients.

Our secondary objective in this case series was to determine if surgery shows similar results to CPAP, taking into account that these are only patients with severe apnea, and that surgery usually tends to be avoided in this kind of patients. We used only 2 criteria to assess the treatment success: the AHI and the nocturia events number (NEN). Our results show that both groups had similar results, regarding age, AHI and nocturia events number prior to treatment. And the results after the use of continuous positive airway pressure devices were comparable to those found 3 months after surgical management with a *p*<0.000 in both groups. The mean AHI decreased from 54.59 to 6.63 in the CPAP group, while in the surgically treated patients decreased from 40.02 to 7.74. The mean NEN decreased from 4.53 to 0.51 in CPAP patients and from 3.78 to 0.7 in surgery patients.

On this study, we included only severe OSAS patients in order to increase the statistical internal validity. New studies are needed in order to determine if our findings can be applied to mild and moderate OSAS as well.

We should keep in mind that we are not comparing CPAP vs any specific modality of surgery. We are comparing it with a multilevel surgical approach, addressing every single anatomical level of obstruction. Our criteria to decide whether an obstructive sleep apnea patient is a good candidate for surgery or not, is not only based only on the AHI, but on the BMI which must be under 30, neck circumference <16 inches, very obvious sites of obstruction in the upper airway and most important: the absence of collapse of the pharyngeal walls. Our findings show the efficacy of surgery in carefully selected OSAS patients, and this efficacy was demonstrated in both the AHI and the NEN, regardless the fact that all of them had severe obstructive sleep apnea.

Pathophysiology of urinary symptoms may not be clear when talking about sleep disordered breathing. The role of atrial natriuretic peptide in the development of these symptoms is increasingly clear. The presence of systemic arterial hypertension and the changes in intrathoracic pressure secondary to the impossibility of introducing air during obstructive apnea events, may lead to an excess of atrial blood volume. This stimulates the production of ANP, along with the levels of hypoxemia, and this explains nocturia, as well as its improvement after treatment.

## CONCLUSION

Systemic hypertension and the changes in intrathoracic pressure during the apneic events are well known consequences of OSAS, and both increase the levels of ANP. This may lead to the need of urination during the night. In subjects with clinical symptoms of obstructive sleep apnea, nocturia should always be part of their clinical assessment. A NEN value of 4-5 may be associated to the presence of severe OSAS, but a thorough clinical evaluation is needed to exclude any other nocturia risk factor.

CPAP remains as the gold standard in the management of sleep-disordered breathing, especially in severe OSAS cases. Nevertheless, with an exhaustive preoperative clinical assessment, selected patients even with severe OSAS, may be candidates for successful surgical management. In our series, CPAP and multilevel surgeries decreased AHI and NEN.

The role of CPAP in the control of nocturia related to sleep-disordered breathing has been previously established. Our findings support the need of nocturia evaluation in OSAS patients, and the value of surgery in accurately selected patients. More studies are required to confirm our conclusions.
